# Report of the intergovernmental panel on climate change: implications for the mental health policy of children and adolescents in Europe—a scoping review

**DOI:** 10.1007/s00787-020-01615-3

**Published:** 2020-08-26

**Authors:** Vera Clemens, Eckart von Hirschhausen, Jörg M. Fegert

**Affiliations:** 1 Department for Child and Adolescent Psychiatry/Psychotherapy, University of Ulm, Steinhövelstr. 5, 89073 Ulm, Germany; 2 Scientist for Future/Foundation Healthy Planet, Healthy People, Berlin, Germany; 3 European Society for Child and Adolescent Psychiatry (ESCAP) Board Member, Head of the Policy Division of ESCAP, Ulm, Germany

**Keywords:** Climate change, Global warming, Mental health, Children and adolescents, Psychological consequences

## Abstract

Climate change is a worldwide challenge. Its consequences do encompass severe threats not only for the existence and somatic health, but also for the mental health of children and adolescents. Mental health can be impaired by three types of consequences. Direct consequences of climate change, such as natural disasters and indirect consequences, such as loss of land, flight and migration, exposure to violence, change of social, ecological, economic or cultural environment. Moreover, the increasing awareness of the existential dimension of climate change in children and adolescents can influence their well-being or challenge their mental health. Consequences of climate change for somatic health may interact with mental health or have psychological sequelae in children and adolescents. Based on the estimates by the United Nations Intergovernmental Panel on Climate Change, we have summarized current data on these differential pathways as to how climate change affects the mental health of children worldwide through selective literature research on Pubmed. Mental health sequelae of direct and indirect consequences of climate change, increased awareness and physical health problems caused by climate change are presented. We give insights into special vulnerabilities of children and adolescents and identify high-risk groups. As the “Fridays for Future” movement has been initiated in northern Europe, we will discuss these results with a focus on children and adolescents in Europe. The results indicate that climate change is a serious threat to children and adolescent mental health. Children´s rights, mental health and climate change should not continue to be seen as separate points; instead, they need to be brought together to address this major challenge determining the future of our children and their descendants.

## Introduction

Climate change is a worldwide challenge that goes along with existential threats especially for future generations—today’s children, adolescents and their descendants. However, climate change will not only threaten the existence of future generations, it may also cause major implications towards the mental health of today’s and future children and adolescents.

**Fig. 1:**
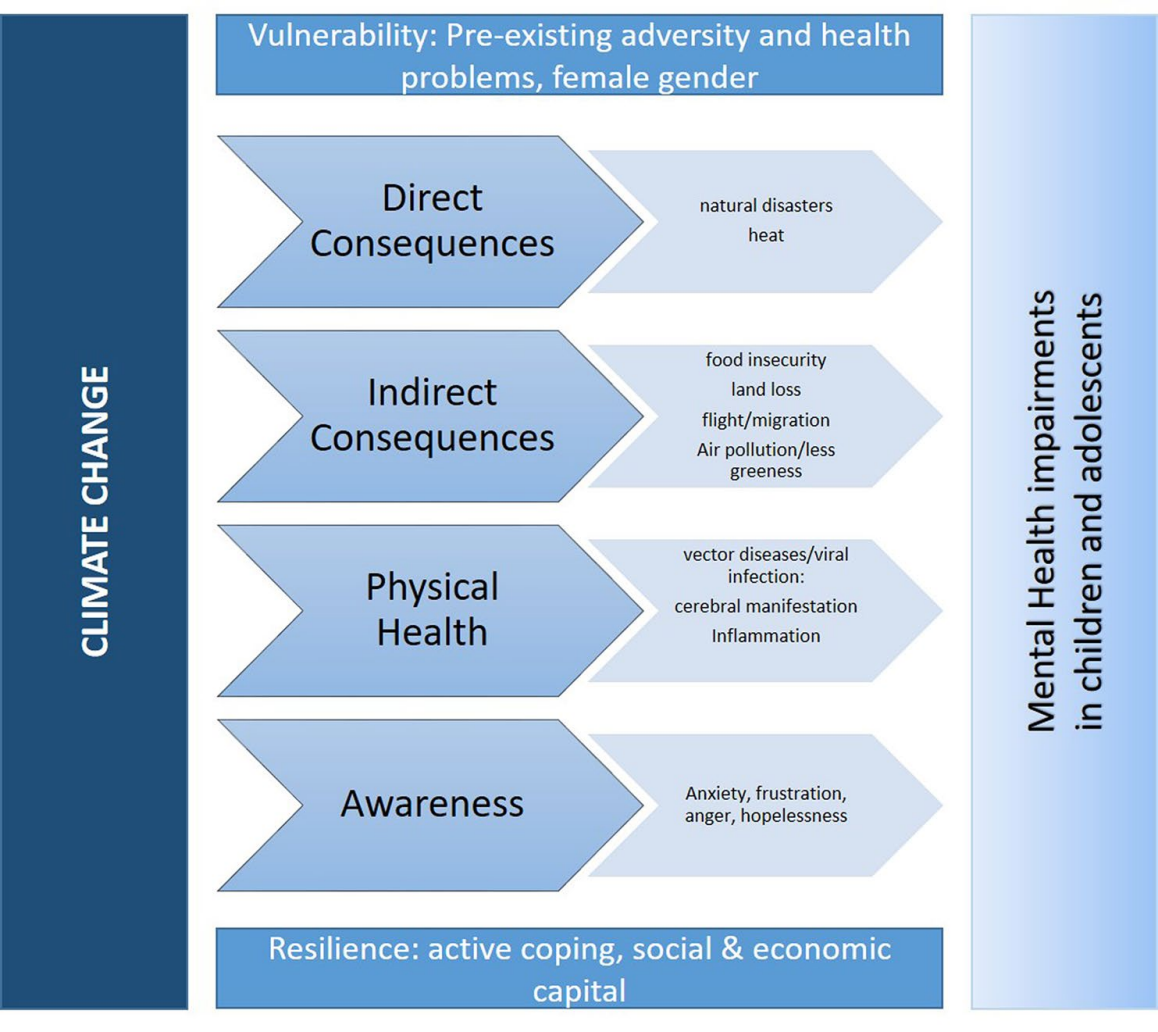
Climate change can affect mental health in directly by, e.g., natural disasters, indirectly by consequences of climate change that affects social, economic and environmental aspects of life and by overarching awareness of climate change and its global consequences. Additionally, physical health problems may impact mental health

Climate change can affect mental health in three ways. Directly, due to natural disasters; indirectly through consequences of climate change that affects social, economic and environmental aspects of life. An example of this would be stress due to crop shortfalls caused by drought. Furthermore, mental health can be impacted by overarching awareness of climate change and its global consequences [1]. As physical health is strongly interwoven with mental health, we will introduce major physical health impairments and possible implications for mental health. Based on information estimated by the United Nations Intergovernmental Panel on Climate Change (IPCC) [2], in this review, we will address all these potential pathways and present current literature with a special focus on European studies (Fig. 1).

The aim of this article is to provide an overview of potential mental health consequences in children and adolescents that may go along with climate change. Due to the complexity of the issues and research questions as method, a scoping review was chosen. A selective scientific literature review on Pubmed was conducted. The search terms were “natural disasters AND mental health AND children”, “heat AND mental health AND children”, “flight AND mental health AND children “greenness AND mental health AND children”, “air pollution AND mental health AND children”, “coping AND climate change AND mental health AND children”. Meta-analysis and systematic reviews were preferred. Single studies were selected if they provided additional information or no systematic approaches were found. Due to the aim of the work, European analyses were preferred. The approach was not systematic. If relevant literature was cited in the literature based on the search terms, it was included when providing additional information. For consequences of food insecurity and physical health problems associated with climate change, several search terms were used to identify literature.

In the main text, we first discuss the direct consequences of climate change on the mental health of children and adolescents, namely due to natural disasters and increased temperature. Additionally, we describe how indirect consequences of climate change can affect us—in detail, consequences due to food insecurity, due to loss of land, due to flight and migration, and due to population in cities. In the next part, we report on how increased awareness can affect the mental health of children and adolescents. Towards the end, we discuss potential consequences for mental health caused by physical diseases associated with climate change.

## Direct consequences

### Consequences of natural disasters

“Increasing warming amplifies the exposure of small islands, low-lying coastal areas and deltas to the risks associated with sea level rise for many human and ecological systems, including increased saltwater intrusion, flooding and damage to infrastructure (high confidence)” [2].

#### Posttraumatic stress disorder

Data on prevalence rates for posttraumatic stress disorder (PTSD) and depression in survivors of natural disasters vary significantly. In two meta-analyses, rates between 1.0 and 60% for PTSD, and 1.6 and 44.8% for depression were reported [3, 4]. This variation may be due to differential impacts of disasters according to their magnitudes and region. Exemplarily, after an earthquake in Abruzzo, Italy, assessing 1,839 children and adolescents, the prevalence of post-traumatic stress (according to Child Behavior Checklist) was 8.4% in the epicenter, 4.0% in the remainder of the earthquake zone and 2.2% in the unaffected area [5]. There is a huge diversity of research methodologies in this field regarding time of assessment and design of studies—including cross-sectional and longitudinal approaches. Based on their systematic review of literature until 2011, Wang and colleagues concluded that the relevance of trauma for development is long lasting, although a peak of symptoms occurs within the first year after exposure. The authors highlight that symptomatology may persist [3]. In a more recent Islandic study, children exposed to a volcanic eruption had more than double the risk for anxiety and worries. Prevalence did not decrease within the next 3 years [6].

Notably, interpersonal trauma is linked with higher PTSD rates and severity [7, 8]. While natural disasters are mainly seen as fate, climate change adds an interpersonal dimension, linked to dealing with generational conflict. This emotional struggle might manifest in higher rates and severity of PTSD.

#### Other mental health problems

Apart from PTSD, after the experience of natural disasters, common psychiatric manifestations among children include internalizing disorders. Elevated rates of social phobia, separation anxiety and psychotic disorders were found [9, 10]. Notably, in a sample of hurricane-exposed children in Puerto Rico, rates for major depression, social phobia, and separation anxiety were more common than PTSD 18 months after disaster [9]. These results were underlined by a meta-analysis by Rubens, Felix and Hambrick who summed up 62 studies including a total of 376,990 participants for internalizing and 27,496 participants for externalizing disorders. The authors found increased rates for internalizing and externalizing disorders after disaster exposure including depression, panic, anxiety, and aggressive behavior, showing a number of mental health problems as sequela of trauma exposure. However, low quality of methodology, e.g., missing of adequate comparison groups, is criticized [11].

Importantly, the sequela of exposure to natural disasters is not limited to childhood and adolescence. Mclean and colleagues assessed 27,129 individuals from a national epidemiologic survey in the US and found increased risks for anxiety and depression in adulthood after the experience of any type of natural disaster before the age of five [12]. The presence of mental illness in parents additionally burdens children and adolescents [13].

#### Risk factors

In the metaanalysis of Rubens, Felix and Hambrick, an exposure-dependent association was demonstrated for other internalizing and externalizing disorders including panic, anxiety, and aggressive behavior. The effect size for non-PTSD internalizing disorders was comparably higher to those of PTSD after disaster exposure [11].

Lai and colleagues summed up data of eight empirical studies, including 8306 children and adolescents and showed that female gender, disaster exposure, negative coping, and lack of social support were significant risk factors for chronic trajectories across several studies [14]. In a meta-analysis including data of 74,154 children, higher death toll, child proximity, personal loss, perceived threat, and distress at time of event were each associated with increased PTS [14]. For depression, in a meta-analysis of 12,890 children, significant predictors were: being trapped during the disaster, experiencing injury, fear, or bereavement during the disaster, witnessing injury/death during the disaster or having poor social support [4]. Focusing on any internal or external disorder or distress, socioeconomic factors such as, the human development index of the country are relevant pointing towards more severe effects in poverty, as Rubens and colleagues concluded in their meta-analysis [11] although a review by Norries and colleagues concludes that for children, family-related factors are primary [15]. Tang and colleagues found in their meta-analysis that prior trauma is a risk factor for the development of depression in children after natural disasters [4]. Additionally to this results, single studies point towards the relevance for other childhood adversities as risk factor for mental health problems after disasters. In a cross-sectional study form Poland, 28 months after a flood lack of parental support, family conflict and overprotectiveness predicted higher PTSD symptom severity [16]. In an Italian study assessing youth after an earthquake, child or maternal history of mental health care prior to the earthquake increased the risk for post-traumatic stress by 7.1 and 4.5 [5]. Notably, these results indicate that the load of previously experienced adversity increases the risk for mental health problems after exposure to natural disasters and weakens each individual’s resilience.

There are a number of studies that provide evidence for higher severity in posttraumatic stress reactions in females [14, 17]. In contrast, Goenjian et al. found in a sample of Nicaraguan adolescents after hurricane Mitch that female gender was not associated with higher PTSD scores if subjective features of exposure (including experience of fear, horror and helplessness) were included into the analyses as confounder. However, as the authors state by themselves, this does not automatically exclude any sex difference but may point towards a more severe subjective experience of fear in females [18]. In their meta-analysis, Tang and colleagues identified female gender as a risk factor for the development of depression after a natural disaster, while this was not the case in children [4]. Rubens and colleagues discussed in their meta-analysis that there are no sufficient studies that take gender into account, which is why they have not assessed the role of gender [11]. Data for age during exposure and PTSD are contradictory. In the single study of Bokszczanin, assessing children and adolescents 28 months after a flood in Poland, younger participants showed more PTSD symptoms [17]. Rubens et al. found no significant moderation effect for age regarding the association of natural disaster exposure and external, as well as internal disorders in adulthood [11], querying the results of single studies such as the above mentioned.

In summary, a high number of studies provide evidence for poorer mental health in children and adolescents who have been exposed to natural disasters. Most of these studies were conducted outside of Europe. Rubens et al. found a stronger association between disaster exposure and internalizing, as well as externalizing problems in medium Human Development Index nations compared to high and very high Human Development Index nations. This points towards more severe effects for children and adolescents in developing nations with limited financial resources [11]. These results suggest the relevance of socioeconomic and cultural factors and show the need for more studies in European samples of children and adolescents after natural disaster exposure. This can help to improve the understanding of direct impact of climate change on European Youth. The data mainly encompass single disasters; whereas, climate change and the experience of multiple disasters become more likely. This may result in higher rates and severity of mental health consequences than in the quoted literature.

### Consequences due to temperature increase

“Temperature extremes on land are projected to warm more than GMST (high confidence): extreme hot days in mid-latitudes warm by up to about 3 °C at global warming of 1.5 °C and about 4 °C at 2 °C, and extreme cold nights in high latitudes warm by up to about 4.5 °C at 1.5 °C and about 6 °C at 2 °C (high confidence). The number of hot days is projected to increase in most land regions, with highest increases in the tropics (high confidence).” [2].

The effect of extremely hot temperatures on mental well-being is evident to any individual; however, there is a lack of literature focusing on the association between mental health in children or adolescents and heat. Global warming goes along with climatic extremes, such as higher temperatures in summer. Majeed and Lee pointed out in a comment that climate change-associated heat itself could impact mental health of children and adolescents [19]. Indeed, data point towards an effect of hot temperature on mental health in children and adolescents. In a study that assessed mental health-related emergency room visits in California in 6- to 18-year olds, the risk for mental health-related emergency room visits augmented by 7.3% per 10 °F (5.6 °C) increase in mean apparent temperature in the warm season [20]. In four US metropolitan areas, a positive association was found regarding crisis support-seeking behavior and high minimum or maximum temperatures among adolescents and young adults [21].

For adults, there is more literature regarding the association of high ambient temperature and mental health. For example, a Chinese study including 20,000 adults found no significant association between mental health and mean temperature but an association between mental health and temperature variability [22]. In an Australian study, a positive association between temperature and hospital admissions for mental and behavioral disorders was seen above a threshold of 26.7 °C, with an increase by 7.3% during heat waves. Illnesses encompassed mental health problems caused by somatic problems, dementia, mood disorders, neurotic, stress related, and somatoform disorders as well as disorders of psychological development. Most interestingly, behavioral and emotional disorders with onset usually occurring in childhood and adolescence (F90–F98) showed a statistically significant decrease in admissions during heat waves compared with non-heat-wave periods in an Australian city [23]. However, it is important to consider the data were derived by a sample above the age of 15 years, not allowing any conclusion for children and adolescents.

The contradictious findings highlight the need for studies assessing the effect of heat on mental health and psychological development of children and adolescents. As climatic conditions are a highly local phenomenon comprising not only ambient temperature but also, among other factors—e.g., humidity, wind, sun, clouds and rain, and temperature variation between day and night, the presented results are hardly generalizable. Thus, the contradictory findings may be partly explained by other not only climate-associated but also cultural and socioeconomic factors such as, e.g., the need to walk long distances during heat, availability of air conditioning and night temperature that may affect sleep. Besides the study in Chinese students, these studies mainly have a retrospective design and only assess mental health problems occurring in contact with the medical system. As access to mental health care and coping with mental health problems differs strongly between countries, the data are hardly comparable.

Heat can also have a significant impact on physical health. Importantly, for adults, psychiatric disorders were shown to be a risk factor for hospitalizations due to heat-related illnesses [24].

Additionally, the ability of children to learn can be impacted by hot temperature. Optimal temperature for learning is around 20 °C but not higher than 23 °C [25, 26]. In a study where elementary classroom temperature in Costa Rica was reduced from 30 to 25 °C, performances in language and logical thinking improved significantly [26].

Therefore, climate change may also impair cognitive development and academic achievements of children and adolescents. Economic aspects may determine how optimal conditions in, for instance, classrooms can be maintained. This may potentially lead to significant disadvantages for some children, in one of the most important dimensions to overcome social injustice: education. However, the existing data are based on few singular studies and further research from different climate zones is needed.

## Indirect consequences

### Consequences due to food insecurity

“Climate-related risks to health, livelihoods, food security, water supply, human security, and economic growth are projected to increase with global warming of 1.5 °C and increase further with 2 °C” [2].

The regular availability of food and clean water is not only an essential prerequisite for physical but also mental health. Literature indicates that food insecurity is a severe threat for the mental health of children and adolescents. Nutrients, including vitamins and fatty acids, are highly necessary for the healthy development and metabolism of the brain [27, 28]. Besides these physical implications, food insecurity results in increased stress for affected children and their families. Stress, in turn, is strongly involved in the pathogenesis of mental health problems, demonstrating another way in which food insecurity can affect the mental health of children and adolescents.

Malnutrition during pregnancy or infancy leads to multiple abnormalities in the central nervous system. Protein malnutrition and deficits in vitamins, iron, fatty acids and zinc were shown to be associated with a lower number of neurons, decreased dendritic arborization and impaired synaptic formation and pruning, myelination deficits, a lack of neurotransmitters and receptor malfunctioning [27]. On the clinical level, in a Taiwanese study of more than 150,000 adolescents, birth at term with low birth weight, an indicator for malnutrition during pregnancy, was associated with lower cognitive performance [29]. In another Taiwanese study including 764,526 children in elementary schools, birth at term with low birth weight was associated with mental disorders [30]. Food insecurity at an older age is associated with cognitive and emotional impairments. In children between 4 and 36 months of age that come from low-income families, food insecurity was associated with a higher risk for developmental problems [31]. In a US study, enlarged food insecurity at the age of 3 correlated with increased behavioral problems [32]. In a study of 6- to 11-year-old US children, food insecurity was associated with poor academic performance as well as psychosocial problems [33]. In a British cohort study of 1,116 families, food insecurity at the age of 5 predicted elevated rates of children’s behavior problems 6 years later [34]. Most importantly, food insecurity encompasses not only the amount of food available, but also quality of nutrition. The quality of food is known to be relevant for mental health. Exemplarily, two European studies point towards the relevance of a varied high quality diet including fresh fruits and vegetables for children’s mental health [35, 36]. Climate change, along with extreme climate, may strongly decrease the variety of plants cultivable in affected regions, threatening not only the amount of available food but also the quality of diet, compromising CNS development and mental health.

### Consequences due to loss of land

“Model-based projections of global mean sea level rise (relative to 1986–2005) suggest an indicative range of 0.26 to 0.77 m by 2100 for 1.5 °C of global warming […]. Sea level rise will continue beyond 2100 even if global warming is limited to 1.5 °C in the 21st century (high confidence). Marine ice sheet instability in Antarctica and/or irreversible loss of the Greenland ice sheet could result in multi-metre rise in sea level over hundreds to thousands of years” [2].

Data on the influence of irretrievable loss of land, due to increasing sea water levels, are scarce. The few existing data deal with loss of land due to temporary natural disasters like floods and hurricanes. The temporary evacuation of homes lead to several losses, including family, peer and community networks in children who experienced a flood in England. The entire daily life was destroyed which leads to severe insecurity and suffering [37]. Affected children experienced distress, anxiety and disillusionment with societal responses. Material and spatial displacement was strongly connected to the experience of social dislocation [38]. Notably, loss of land can not only lead to societal insecurity, but also lead to economic instability, resulting in family pressure, affecting family structure and family harmony. Together, this can lead to a severely disrupted sense of belonging [39]. Even though data are not specific for children, capital—in the form of social and economic capital—is of major importance for mental health effects of flooding [40], meaning that subjects that are already disadvantaged are at higher risk of more suffering. However, land loss due to climate change may differ strongly from land loss in the mentioned studies. Size of the affected areas could be much larger, the perspective of being able to return could be lacking and the risk of such experiences being repeated could increase. Therefore, the studies above may only partly reflect mental health problems caused by land loss due to climate change.

### Consequences due to flight and migration

“Climate models project robust differences in regional climate characteristics between present-day and global warming of 1.5 °C, and between 1.5 °C and 2 °C. These differences include increases in: mean temperature in most land and ocean regions (high confidence), hot extremes in most inhabited regions (high confidence), heavy precipitation in several regions (medium confidence), and the probability of drought and precipitation deficits in some regions (medium confidence)” [2].

#### Consequences of flight

The consequences of flight and migration for mental health are widespread and have been shown numerously. Refugee experience can be divided into three phases: the preflight phase, the flight phase and the resettlement [41]. In the preflight phase, due to collapse of social and economic structures, daily structure gets lost. Leisure time activities break down, access to education is limited or non-existent, and important interpersonal relations such as with family members and peers, may get lost as they may have fled before. This phase and the consecutive mental health effects are strongly linked to the reason of the flight (see previous chapters). The flight phase is characterized by huge uncertainty. This encompasses basic needs such as nutrition and shelter, but also separation from parents and caregivers [41]. In addition, children and adolescents at flight are at high risk of violence, abuse and trafficking. In refugee camps, children and adolescents are at a high risk of violence, discrimination and harassment. If families are not separated, family structures risk being severely challenged [42]. During resettlement, adaption towards the new setting including language and culture is necessary. In a systematic review of the literature, Fazel and colleagues showed that children and adolescents suffer from the loss of their homeland, family, friends, and material possessions. This can lead to feelings such as guilt, anger, and ambivalence. Moreover, challenges can include adaptation processes to the new setting, racial discrimination, and complex legal immigration processes that go along with great uncertainty for their own future as well as the future of family and friends [43].

#### Flight-associated mental health problems

Minor refugees are at high risk for the development of mental health problems. In a systematic review including studies from 1990 until 2017, rates for PTSD were shown to vary in dependence of the study between 19 and 53%, rates for depression between 10 and 33%, anxiety disorders between 9 and 32%, and emotional and behavioral problems between 20 and 35% [44]. Even though the data are not specific for climate change-associated flight and migration, exposure to violence was shown to be a major risk factor, while stable settlement and social support in the host country are protective factors for the mental health of minor refugees [43]. Importantly, for flight due to climate change, where the experience of pre-flight violence may be less likely compared to, e.g., war, violence experienced during migration was also shown to be associated with PTSD symptoms [45]. Remarkably, there are data that indicate that parental exposures to traumatic events are more strongly associated with children’s mental health problems than children’s own exposures [43], indicating the high relevance of parental mental health for minor refugees. A group at risk particularly for mental health problems is that of unaccompanied refugees [44]. In an assessment of 1,294 refugee adolescents living in Belgium, 10% of which were unaccompanied, it was shown that unaccompanied refugees are at higher risk both for mental health problems and to have experienced traumatic events [46], highlighting the vulnerability of this group.

Taken together, flight may be one of the most important climate change-associated threats for mental health in children and adolescents. Although there are a high number of studies assessing the effects of flight on children and adolescents, most frequent flight causes are war or war-like conditions. Consequently, the transferability of the results presented here regarding mental health consequences of flight is limited. Moreover, there is a huge variability in prevalence rate due to different assessments of mental health problems, samples and study designs [44]. Nevertheless, the data point towards significant mental health impairments for affected children and adolescents [47].

### Overpopulation in cities: Less greenness, more air pollution

Living in big cities is associated with higher rates of mental health problems in children and adolescents [48, 49], even though the detailed mechanisms are not yet known. Social factors that may mediate this association are higher violence exposure, less social coherence and residential instability [50]. Two additional factors closely related to climate change are exposure to nature/green spaces and air pollution.

#### Greenness

Natural environments including green spaces are associated significantly with positive attributes such as physical activation, creativity and relaxation. Hence, they are discussed to contribute significantly to mental health.

In their systematic review on the impact of green space exposure on children’s and adolescents’ mental health, Vanaken and Danckaerts concluded a beneficial association between green space exposure and children’s emotional and behavioral difficulties. This was particularly the case with hyperactivity and inattention problems, while only limited evidence suggested a beneficial association with mental well-being in children and depressive symptoms in adolescents and young adults [51]. An inverse correlation between green with deficits in visuo-motor and language development was shown. A positive correlation with progress in working memory and superior working memory, and a reduction in inattentiveness was found, suggesting enhanced neurocognitive development in the case of higher greenness exposure. [51]

In a study from Spain of 253 children, lifelong exposure to greenness was positively associated with gray matter volume in regions that were associated with cognitive test scores, working memory and reduced inattentiveness [52] Vanaken and Danckaerts discuss the role of physical activity, fewer air pollution and social interaction as potential mediators. However, time spent in the green and proximity may be an important confounder that was mostly not assessed, and may be relevant for this association [51].

In a cross-sectional Lithuanian study of 1,468 children aged 4–6, farther residential distance from city parks was associated with more mental health problems only in children whose mothers had a lower education level [53]. This points towards a complex interplay between greenness exposure and socioeconomic status. However, in their systematic review, Vanaken and Danckaerts point out that the association between mental health of children and adolescents is resistant to adjustment for demographic and socio-economic confounders [51].Air pollution

Another factor linked to city overcrowding that may harm mental health is air pollution. Air pollution is linked to oxidative stress and inflammatory activation [54, 55], both centrally involved in the development of psychiatric disorders [56, 57]. Additionally, air pollution in cities is usually linked to high traffic and noise, additionally impacting mental health. Studies point towards a negative association between air pollution and mental health.

In a large UK cohort Study, exposure to annualized PM_2.5_ and NO_2_ at the age of 12 was associated with increased risk for depression at the age of 18, but not with concurrent mental health problems at the age of 12 [58]. In a Swedish cohort study, children and adolescents living in areas with a higher air pollution concentration were more likely to have a dispensed medication for a psychiatric disorder during 3.5 years of follow-up [59]. Based on the cross-sectional and prospective studies, air pollution is linked to behavioral problems [60–63] and emotional problems [64] in children. Furthermore, there is a growing body of evidence pointing towards an association between exposure of children to air pollution and ADHD [65]. A nationwide case–control study of 15,387 children with autism spectrum disorder (ASD) in Denmark found an increased risk for ASD in case of air pollutant exposure in early infancy but not during pregnancy [66]. In a Swedish register-based cohort study including 48,571 children, associations between air pollution exposure during the prenatal period and the risk of developing ASD were found [67].

However, critics point out that in many of the studies assessing the consequences of air pollution, definitions regarding air pollution are imprecise and exposure indicators not sufficiently localized [66]. In addition, it is important to keep in mind that living conditions are associated closely with socioeconomic status, which again is a central determinant for mental health. Even though the majority of the literature shown adds parental education and income as confounder, the interplay between socioeconomic status, living conditions and mental health is highly complex, and bias cannot be excluded.

### Mental health impairments due to awareness of climate change and global consequences

“Human activities are estimated to have caused approximately 1.0 °C of global warming above pre-industrial levels, with a likely range of 0.8 °C to 1.2 °C. Global warming is likely to reach 1.5 °C between 2030 and 2052 if it continues to increase at the current rate. (high confidence)” [2].

In the European countries like Germany, Austria, Switzerland and the UK, the majority of young people perceive climate change as a big problem [68]. During the last few years, awareness for climate change in adolescents and young people has grown in Europe. One indicator is the “Fridays for Future” movement that started in summer 2018 and has led to a massive involvement of adolescents and young people participating in the public discussion regarding climate protection. This increasing awareness may also have negative consequences for the mental health of children and adolescents. A study found that Swedish 12-year olds recognize climate change as a stressor [69].In a study on Australian adolescents, the experience of drought was associated with more intellectual engagement with climate change and higher emotional distress [70]. Consequently, this study does not only point towards an impact of climate change-related worries on mental health of adolescents, but also suggest that direct experience of climate change-associated problems may increase these problems. In a US study, interviews with children aged 10–12 revealed fear, sadness, and anger in more than 80% of participants when discussing their feelings about environmental problems. Additionally, the majority expressed apocalyptic and pessimistic feelings about the future of the planet [71]. These negative feelings may lead to grief, anxiety and hopelessness, which in turn may impact their mental health.

Notably, children and adolescents seem to be less fatalistic about combating climate change compared to adults [68]. Maria Ojala assessed how 12-year olds cope with climate change. The results indicate that problem-focused coping was positively correlated with general negative affect while children with a high degree of meaning-focused coping were less likely to experience negative affect [72]. This points towards the relevance of self-efficacy for the coping with climate change. Self-efficacy is a key factor for coping with problems and the prevention of mental health problems [73]. Young people feel powerless in the face of climate change and think that personal action would not make a difference [68]. Besides the feelings of anxiety and sadness, this lack of self-efficacy may be one major climate change awareness-associated threat for the mental health of children and adolescents.

### Physical health

“Risks from some vector-borne diseases, such as malaria and dengue fever, are projected to increase with warming from 1.5 °C to 2 °C, including potential shifts in their geographic range (high confidence).” [2].

Physical health is a central determinant of mental health. On the other hand, mental health is a central determinant for somatic health as several psychiatric illnesses are linked to less well health behavior [74]. There are common pathways that may implicate both physical and mental health problems. These pathways include the activation of the stress system and the inflammatory system. The most common causes for mortality in Europe, including cardiovascular diseases and cancer, are linked to alterations in the stress system and have a strong inflammatory component [75–78]. The same is known for the most common neuropsychiatric disorders including, e.g., affective disorders, post-traumatic stress disorder and schizophrenia [79–81].

Importantly, climate change is thought to be an important factor in the worldwide increase of vectors, consequent disease vectors and the expansion in Europe including malaria, zika, dengue and leishmaniosis, Lyme Disease and West Nile Virus [1, 82]. Outbreaks of these vector diseases may lead to increased awareness and anxiety in the population, as well as immune activation in affected children and adolescents that may contribute to mental health problems. Additionally, several of these diseases have cerebral consequences. Zika infection during pregnancy is linked to microcephaly and extensive brain damage in children [83]. Zika infection during adolescence is discussed to be associated with neurological complications and cognitive deficits [84]. The West Nile virus infection goes along with increased risk for depression [85]. In a study among Malawi schoolchildren, surviving cerebral malaria was associated with neurocognitive deficits [86]. In children under the age of 5 in Uganda, cerebral malaria or severe malarial anemia was associated with increased internalizing and externalizing behavioral problems [87]. In a recent review, leishmaniosis was shown to be associated with mental health impairments [88]. The data point towards an effect on climate change-associated increase of vector diseases—not only by increased stress and by a general activation of the immune system, but furthermore by direct effects of these diseases on the mental health of children and adolescents.

## Discussion

Climate change is a major challenge for the future. In the words of former UN Secretary-General Ban-Ki Moon “Ours can be the first generation to end poverty—and the last generation to address climate change before it is too late.” Regarding the overwhelming number of scientific reports that confirm ongoing climate change and its devastating, self-reinforcing consequences, this gives a glimpse on the pressure and the challenges the future generation will have to cope with. The way in which we—today’s adults—will act to prevent climate change and will respect today’s children and adolescents that make their voice heard, e.g., in the “Fridays for Future” movement founded in Europe, will determine how large future generation conflicts will develop and how we let today’s children and adolescents participate and cope with climate change actively.

The above-summarized data indicate that climate change can lead to major impairments of the mental health of children and adolescents. This encompasses PTBS and other direct consequences of climate change, as well as risks linked to flight and migration, changes in the ecologic, social, cultural and political environment, the awareness of climate change and somatic illnesses. Depending on the decisions that will be made in the next decade, children and adolescents across the world may be affected by the consequences of climate change.

Existing literature was characterized by a high heterogeneity regarding samples, assessment and design. If systematic reviews or meta-analysis were available, results showed wide ranges of, e.g., prevalence. Natural disasters and flight can vary significantly in regards to personal and local circumstances. However, more international and systematic approaches that include detailed circumstances such as—e.g., for flight—flight reasons, social and economic capital, gender, age, experiences before, during and after flight, but also cultural and societal aspects and standardized assessments of mental health problems are needed. Moreover, a lack of prospective studies assessing the relevance of long-term sequelae not only for affected individuals but also for society can be seen.

It is important to point out that there is lack of data that specifically target the question of mental health effects of climate change in children and adolescents, especially when information for specific regions such as Europe is needed. Thus, our review includes a lot of literature that does not directly target climate change but related topics such as mental health sequelae of flight and migration due to other reasons and the effect of singular natural disasters, which may reflect the future challenges of climate change only in a very limited way.

Moreover, it is important to consider that the ecological crisis includes much more than climate change., Mental health of children and adolescents may also affected by the loss of biodiversity and pollutants such as plastic [89], which was not included in the scope of this review.

Regarding the awareness of climate change, which may be one of the first mental health sequelae, nearly no data were found. Those data found were mainly from Europe—yet a region characterized by high socioeconomic and climate security. Future studies should focus on children and adolescents living in places where the threats of climate change are much closer, such as small insular states. The fact that future natural disasters may be perceived as consequences of climate change and thereby “man-made” instead of guiltless fate may significantly affect the consequences for mental health.

Although studies that target the effect of climate change on children and adolescents specifically are scarce, the existing data as well as the conclusions that can be drawn from the transfer of literature on related topics, point towards severe mental health impairments as a consequence of climate change. There is a need to take action to address this issue. Easily accessible interventions that aim to target specific groups such as fled children and adolescents need to be further developed. Programs that strengthen social and professional support for, e.g., children affected by natural disasters need to be built.

### Pre-existing adversities are a risk factor

Besides treatment, action needs to be taken for the prevention of mental health consequences. Resilience refers to the ability to cope well with stressors and thus an interaction between an individual and a stressor. Childhood adversity is a central factor for the resilience of individuals [90] while the cumulative load of adverse childhood experiences leads to an increase of mental health problems [91]. Importantly, much of the shown literature indicates that mental health consequences will particularly affect children and adolescents who are already disadvantaged—those with low or no social support by families and peers, those from families with low socio-economic status and those already suffering from mental health problems. Consequently, protecting children and adolescents from adversity and enabling them to grow up in a supportive, non-violent environment to strengthen their resilience to cope well with the challenges of climate change is an important approach to prevent mental health problems. This should be a priority of any child-related mental health policy.

### Support of active coping

30 years after the signing of UN Convention on the Rights of the Child (UNCRC), child rights-based approaches must be implemented to protect children and adolescents from consequences of climate change. The rights-based sustainable development goals (SDGs), adopted by all United Nations Member States in 2015, define how states are expected to frame their development agendas and political policies over the next 15 years. Goal 16.2 aims to end all forms of violence against children and gives renewed impetus towards the realization of the right of every child to live free from fear, neglect, abuse and exploitation [92].

The literature presented demonstrates how important active coping is for mental health—just as is social support. Movements such as “Fridays for future” show that young people take their responsibility seriously—especially because, from their point of view, adults do not. Greta Thunberg, the 17-year-old climate activist from Sweden, addressed the 2018 UN climate change summit: “Since our leaders are behaving like children, we will have to take the responsibility they should have taken long ago” [93]. This reflects a reversal of roles, which may result in both—empowerment and sense of community among young people, but at the same time loss of confidence into the world of their parents.

The support of active coping of youth may help to prevent the consequences of climate change. Most importantly, if adults do not take action, there is a serious risk that blame and accusations will aggravate the beginning generational conflict, wider divisions in society will provide fertile ground for violence and the feeling of power- and helplessness will exponentiate the already described risks for the mental health of children and adolescents.

It was recently suggested that child and adolescent psychiatry training programs should integrate climate change as a required curriculum topic [94]. Standing together with today’s children and adolescents who claim their right for a healthy future in international movements such as “Fridays for Future” is a major task for all organizations that aim to protect and strengthen mental health of children and adolescents. The “Psychologists/Psychotherapists for Future” are professional psychologists and psychotherapists who support the “Fridays for Future” movement officially and feel responsible for contributing expertise to manage the major challenge of climate change [95]. Now, it is up to child and adolescent psychiatrists and psychotherapists to assume their responsibility and consider aligning in a similar way with today’s children and adolescents.

Taken together, climate change is a major challenge for the mental health of future generations. Prevention of violence against children, mental health problems and climate change cannot be seen separately anymore but have to be brought together to address this major challenge that will determine the future of our children and their descendants.
